# Comparative analysis of whole-genome sequencing of tumor and cfDNA in a neuroblastoma patient: a case report

**DOI:** 10.3389/fonc.2025.1569520

**Published:** 2025-05-02

**Authors:** Susanne Fransson, Kleopatra Georgantzi, Anna Djos, Jennie Gaarder, Johanna Svensson, Vimala Anthonydhason, Per Kogner, Tommy Martinsson, Ganesh Umapathy

**Affiliations:** ^1^ Department of Laboratory Medicine, Institute of Biomedicine, Sahlgrenska Academy, University of Gothenburg, Gothenburg, Sweden; ^2^ Childhood Cancer Research Unit, Department of Women’s and Children’s Health, Karolinska Institutet, and Pediatric Oncology, Astrid Lindgren Children’s Hospital, Karolinska University Hospital, Stockholm, Sweden; ^3^ School of Biomedical Sciences and Pharmacy, College of Health, The University of Newcastle, Newcastle, NSW, Australia

**Keywords:** circulating cell free DNA (cfDNA), whole genome sequencing (WGS), MET (c-MET), liquid biopsy, neuroblastoma

## Abstract

High-risk neuroblastoma (NB) poses significant challenges in pediatric oncology due to its resistance to conventional therapies, leading to relapse and poor prognosis. Copy number variations (CNVs) are strong prognostic factors in NB, prompting exploration into alternative methods for CNV profiling. We conducted whole-genome sequencing (WGS) of the circulating cell-free DNA (cfDNA) from a patient with NB and compared the WGS of the primary and relapsed tumor tissue. Our analysis revealed concordance between the somatic single nucleotide variants (SNVs), insertions and deletions (indels), and CNVs identified in the cfDNA and tumor WGS. Notably, WGS detected numerical chromosome imbalances, large and focal structural aberrations including amplifications in *MYCN*, *CDK4*, and *MDM2*, using low-input cfDNA. Furthermore, additional variants unique to the cfDNA, such as the rare *MET* (p.R970C) variant, were identified, possibly representing sub-clonal populations or variants present at metastatic sites. In conclusion, WGS analysis of cfDNA offers a noninvasive, cost-effective, rapid, and sensitive alternative for CNV profiling in patients with NB. This approach holds promise for improving prognostication and for guiding personalized treatment strategies in NB.

## Introduction

Neuroblastoma (NB) represents a formidable challenge in pediatric oncology. It is characterized by its heterogeneous clinical behavior ranging from spontaneous regression to aggressive metastatic disease (1–4). NB accounts for a significant portion of childhood cancer mortality, particularly in patients with high-risk disease who exhibit resistance to conventional therapies and a propensity for relapse (1–4). NB is also known for its genomic complexity, with different copy number variations (CNVs) as strong prognostic indicators influencing the disease outcome (1–5).

In recent years, the advent of genomic technologies has revolutionized cancer research and clinical practice, offering insights into the molecular underpinnings of tumorigenesis and thereby potential therapeutic targets. Whole-genome sequencing (WGS) has emerged as a powerful tool for comprehensive genomic profiling, enabling the detection of somatic mutations, structural rearrangements, and CNVs across the entire genome (6–8). However, traditional methods of obtaining tumor DNA for WGS or other genomic analyses, such as tissue biopsy, are invasive. Moreover, a biopsy may not always capture the full genomic landscape of the disease, particularly as tumor heterogeneity and distant metastasis are present in many patients with NB. Another obstacle present in some cases is the anatomical location of the tumor, which can make them inaccessible for biopsy (9–12); in other cases, open surgical biopsy could delay clinical management and the start of necessary therapy (13–15).

In contrast, liquid biopsies offer a minimally invasive alternative for genomic analysis, leveraging the presence of circulating cell-free DNA (cfDNA) shed into the bloodstream by tumor cells (16, 17). This is a convenient approach that enables repeated sampling over the course of treatment, thereby providing real-time insights into the tumor dynamics and evolution (17–19). Several studies have demonstrated the possibilities of cfDNA analysis in profiling genetic alterations in various cancer types, including breast, lung, and colorectal cancers (20–23). Recent studies have demonstrated the utility of cfDNA in NB, with varying concentrations observed across disease stages and risk groups (24). While targeted sequencing has been widely used to profile cfDNA in NB, WGS remains underutilized despite its potential to provide a more comprehensive view of genomic alterations (25, 26). This study leverages WGS to compare the cfDNA and tumor DNA in a relapsed patient with NB, highlighting the potential of liquid biopsy for noninvasive genomic profiling in pediatric cancers.

Despite its promise, the clinical value of cfDNA analysis in NB is still relatively unexplored. Herein, we present a study aimed at evaluating the feasibility and utility of WGS analysis on cfDNA as a noninvasive alternative to tumor biopsies as the DNA source for CNV profiling in NB. We compare the genomic alterations detected in the cfDNA with those identified in the tumor tissue, leveraging advanced molecular techniques to characterize the genomic landscape of NB and to identify potential therapeutic targets. Our study demonstrates the successful application of WGS for the detection of CNVs and single nucleotide variants (SNVs) in the cfDNA from a patient with NB. These findings suggest that cfDNA WGS holds promise for the evaluation of treatment response, clonal heterogeneity, and early detection of relapse in NB, potentially guiding clinical decisions regarding NB treatment.

## Materials and methods

See **Supplementary Material 1** for an extended description of the methods used.

## Results

### Patient information and clinical findings

**Figure 1:**
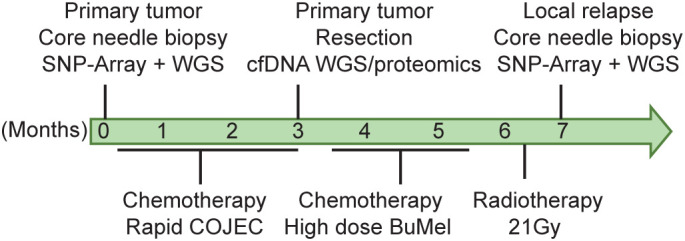
Patient clinical history. Timeline depicting patient follow-up, biopsy, and initial diagnosis. *[C]*, cisplatin; *[O]*, vincristine; *[J]*, carboplatin; *[E]*, etoposide; *[C]*, cyclophosphamide (known as COJEC); *BuMel*, busulfan and melphalan.

The patient, a boy, was diagnosed with high-risk NB at the age of 2 years and 1 month. He initially presented with a 14 cm × 10 cm × 10 cm *MYCN*-amplified tumor localized on the right adrenal gland with overgrowth in the right kidney and with local lymph node metastases in the abdomen. The tumor, INRG stage L2, was crossing the midline, compressing the liver and dislocating and compressing the abdominal aorta and the inferior vena cava (27). At the time of diagnosis, the bone marrow was negative for NB cells and the tumor did not show any uptake on MIBG (meta-iodobenzylguanidine). FDG-PET was not performed. After the investigational workup, treatment was started according to the SIOPEN HR-NBL1 protocol, with partial response (4.3 cm × 3.6 cm × 3.8 cm) to the induction chemotherapy (28). The tumor and the lymph node metastases were removed surgically without any complications 3 months after diagnosis. According to the pathology report, the tumor was completely resected and showed >90% necrosis and <10% viable cells. At 2 weeks after the operation, the patient underwent high-dose chemotherapy with BuMel (busulfan and melphalan) followed by stem cell reinfusion. Proton radiotherapy to the initial tumor bed was administered with a total dose of 21 Gy. A short time after completion of radiotherapy, clinical deterioration was observed, and the disease progressed with metastases in the liver, the lungs, bone marrow, and the right kidney, near the site of the primary tumor. The treating physicians applied for compassionate use of the MDM2 inhibitor; however, due to the rapid progression, the treatment was never initiated. Ad mortem was 8 months from the initial diagnosis (**Figure 1**).

### Genetics of the patient

**Figure 2:**
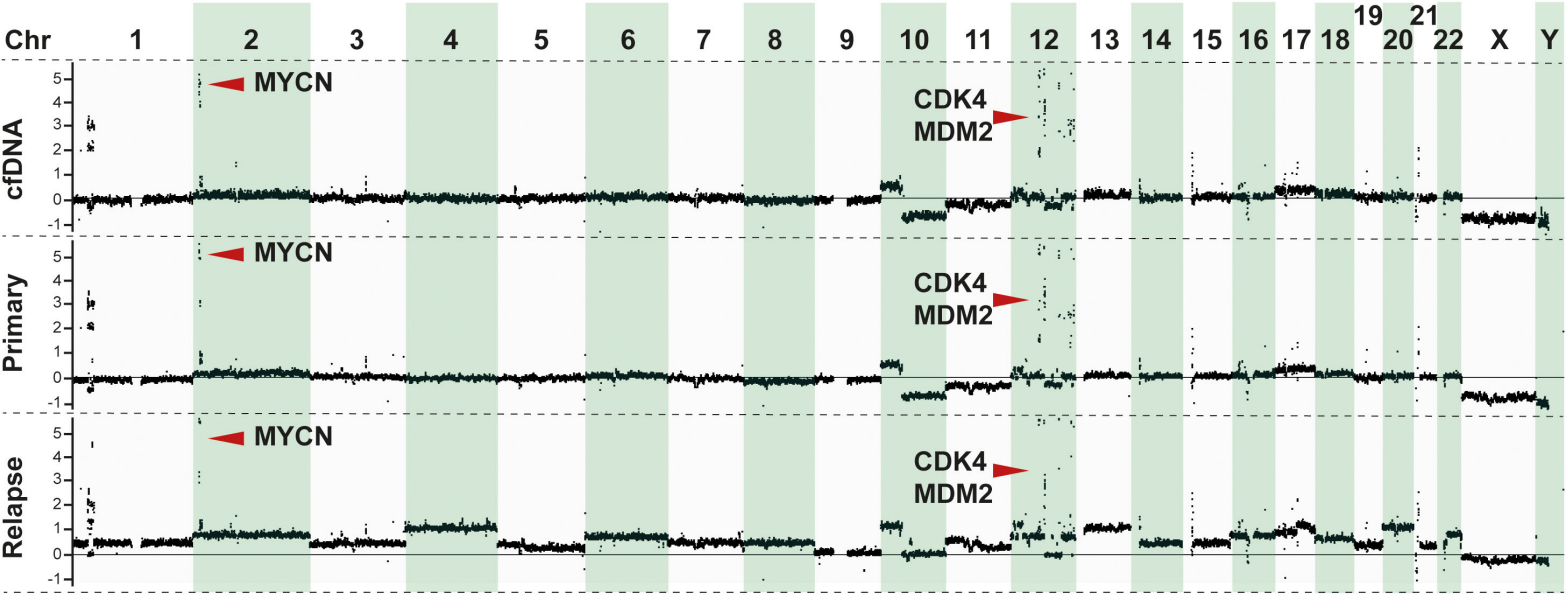
Genomic analysis of tumor samples. Copy number profiling derived from normalized reads from whole-genome sequencing (WGS) showing the genomic profiles of the patient from cfDNA (*upper panel*), primary tumor (*middle panel*), and relapse (*lower panel*). *Y*-axis shows the copy number change in relation to the diploid genome (set as zero) as inferred by the Canvas tool (49).

DNA extracted from the tumor biopsy, which was retrieved from the primary tumor at the time of diagnosis, and DNA from a local relapse sampled 7 months after the diagnosis, together with constitutional DNA extracted from blood lymphocytes, were analyzed using WGS with average sequencing depths of 59×, 104×, and 37×, respectively (**Supplementary Table S1**). Copy number profiling by WGS and SNP microarray showed concordant findings and indicated a complex genome with amplification of multiple regions on chromosomes 1, 2, and 12 including *MYCN*, *CDK4*, and *MDM2*, together with an interstitial deletion on 1p, deletion of 10q, gain of 10p and 17q, and a small focal homozygous deletion affecting *PTPRD* on chromosome 9 (**Figure 2**; **Supplementary Figures S1A, B**), with addition of 11q deletion and 22q gain and several numerical alterations at the time of relapse. WGS revealed a total of 19 and 147 somatic, non-synonymous SNVs with variant allele frequency (VAF) ≥10% in the primary and relapsed tumor material, respectively, of which 16 were shared between the two tumor specimens (**Supplementary Figure S2A** and **Supplementary Table S2**). A total of 107 structural variants (SVs) were common to the two tumor samples, with an additional 131 SVs unique to the tumor at the time of diagnosis and 225 SVs unique to the tumor at the time of recurrence. The vast majority of called SVs were associated with the amplified regions (**Supplementary Figure S2A** and **Supplementary Table S3**). No alterations in association with *ALK, TERT, ATRX, TP53*, or any of the RAS-genes were detected. Germline analysis indicated no underlying genetic causes for NB.

### Comparison of plasma cfDNA WGS *versus* tumor DNA WGS

The cfDNA extracted from the plasma of the patient retrieved at the time of tumor resection was subjected to WGS, reaching an average coverage of 15× (**Supplementary Table S1**). The analysis aimed to identify somatic mutations, structural variations, focal copy number amplifications/gains and deletions, and other genomic alterations that could provide insights into the genomic landscape of the tumor and potential therapeutic targets.

The cfDNA-generated copy number profile was more similar to that at diagnosis than at relapse (**Figure 2**), including the amplicon on chromosomes 2 and 12 (**Supplementary Figure S1B**). Amplifications, in particular encompassing the *MYCN* locus, are known to infer poor prognosis in patients with NB (29–31). Accurate assessment of *MYCN* is thus of utmost importance in the genetic workup of the primary tumor as it may directly impact treatment decisions. Importantly, in this study, amplification in *MYCN* observed in the tumor DNA samples was also detected in the cfDNA (**Figure 2**). Furthermore, chromosome 12q amplicons containing *CDK4* (12q14.1) and *MDM2* (12q15) were detected both in the cfDNA and the tumor tissues (**Figure 2**; **Supplementary Figure S1B**), consistent with previous findings associating these co-amplifications with poor prognosis in patients with NB (32, 33).

**Figure 3:**
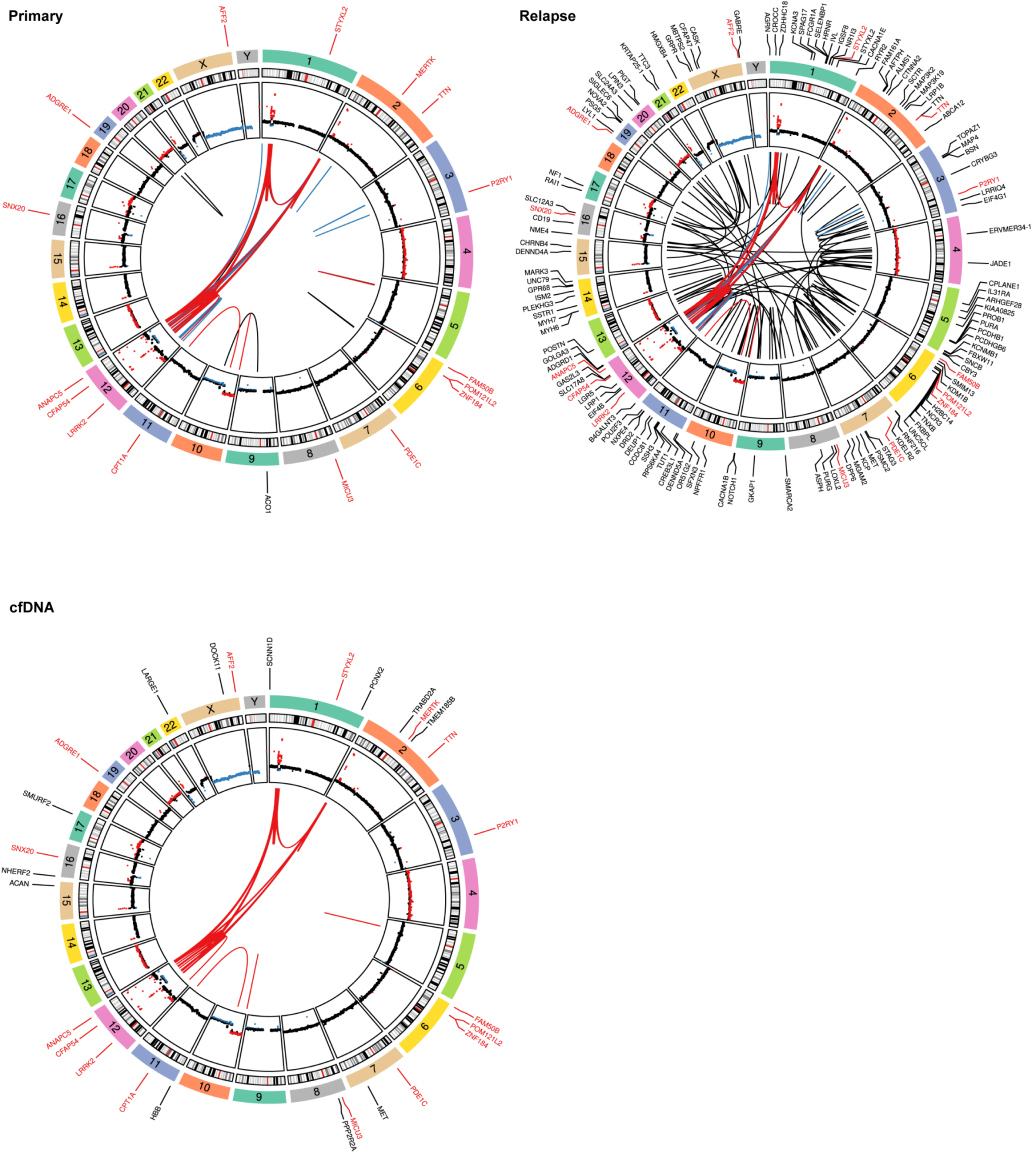
Mutational spectra and structural variants. Circos plots showing structural variants (SVs), copy number variations (CNVs), and somatic single nucleotide variants (SNVs). The copy number plots calculated based on the coverage ratio between the tumor or cell-free DNA (cfDNA) and the corresponding normal tissue are shown on the *inner circle*, with gain of genomic material indicated in *red* and loss of genomic material indicated in *blue*. The *lines within the inner circle* indicate structural variants within and between chromosomes, while the genes affected by somatic SNVs are shown *outside the outer circle*. Alterations shared between the tumor and cfDNA are indicated by a *red font* or *red lines*, alterations shared between primary and relapse are indicated in *blue*, while variants unique to a sample are indicated in *black*. For clarity, only the SVs that are shared between cfDNA and the tumor are depicted in the Circos plot for cfDNA.

Subsequently, we investigated somatic mutations, which provided additional information regarding the heterogeneity of the primary tumor. Indeed, additional genomic alterations were observed in the cfDNA compared with the tumor DNA. Notably, pathogenic or likely pathogenic missense mutations were identified in the cfDNA sample, including SNVs that are consistent with the WGS findings of the primary tumor (**Figure 3**; **Supplementary Figure S2A** and **Supplementary Table S2**). Interestingly, an additional missense variant not detected in the tumor WGS was identified in *MET* (NM_000245.4: c.2908C>T, p.R970C), which might represent a sub-clone (**Figure 3**; **Supplementary Table S2**). Although cfDNA may not be an optimal source for the detection of SVs due to short fragments and the fraction of circulating tumor DNA, still a substantial part of the SVs detected in the primary tumor could be confirmed in the cfDNA (**Figure 3**; **Supplementary Table S3**). Additional SVs unique to the cfDNA were also called (**Supplementary Figure S2B**); however, due to the above-mentioned limitations, these are more ambiguous.

### Protein analysis of the tumor material

**Figure 4:**
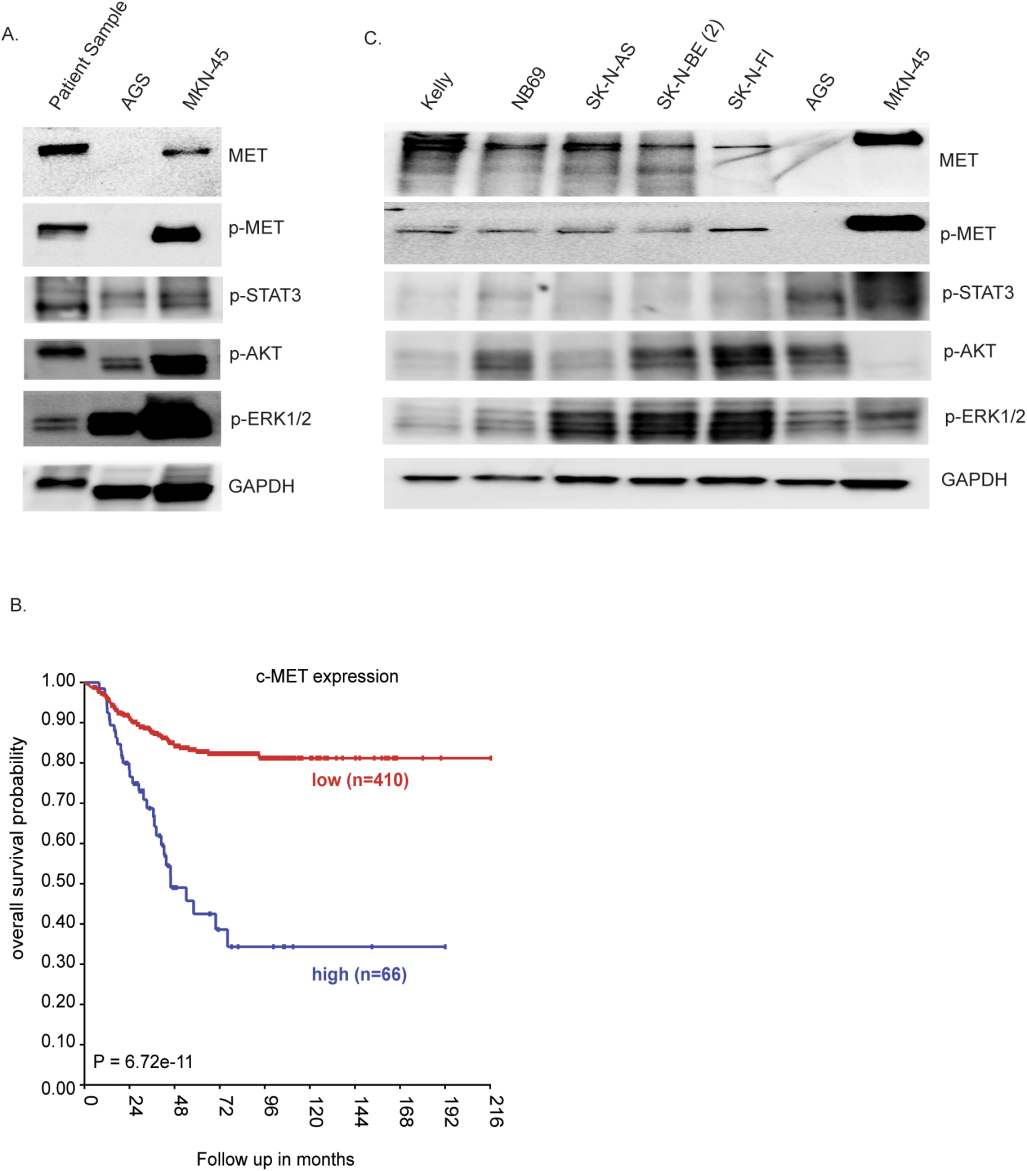
Proteomic analysis of the tumor resection sample. **(A)** Immunoblotting for the indicated proteins in lysates from the gastric cell lines AGS and MKN-45, as reference, and the patient tumor lysate. **(B)** Kaplan–Meier event-free survival curves of the neuroblastoma (NB) cohort Kocak-649 patients stratified according to MET expression. Patients with higher expression are highlighted in *blue*, whereas those with lower expression are highlighted in *red*. The log-rank test *p*-values are indicated. **(C)** NB cell lysates analyzed on SDS-PAGE followed by immunoblotting for MET, phospho-MET, p-STAT3, p-AKT, pERK1/2, and GAPDH as the loading control.

Due to the presence of the *MET* variant in the cfDNA, we wanted to determine whether the patient’s tumor biopsy sample possesses MET activity. To investigate this, fresh frozen tumor tissue from the primary tumor resection was processed for proteomic analysis. In this experiment, we used the gastric cell lines AGS (non-amplified MET) and MKN-45 (amplified MET) as negative and positive controls, respectively, for MET activity (34). Immunoblotting indeed showed detectable protein expression levels of MET, as well as phospho-MET (Y1234/Y1235) (**Figure 4A**), which indicate active MET protein. Signaling pathways, including STAT3, AKT, and ERK, were also activated in the patient sample, although much weaker than those in the MKN-45 cell line with MET amplification (**Figure 4A**). Thus, our proteomic analysis indicates that both MET and its downstream targets might be activated in this NB patient sample. Investigation of an earlier published NB cohort (Kocak-649 patients) (35) showed that an increase of the *MET* gene expression is associated with poor prognosis in NB (*p* = 6.72 × 10^−11^ and *p* = 0.02 respectively) (**Figure 4B**). To investigate the presence of phosphorylated MET, a panel of five NB cell lines [Kelly, NB69, SK-N-AS, SK-N-BE (2), and SK-N-FI], which represent a range of aberrant genetic backgrounds found in NB tumors, although without any MET aberrations (36), were examined. All of the tested NB cell lines expressed MET and displayed detectable p-MET protein levels (**Figure 4C**), suggesting that these cell lines have a basal activation level of MET. In contrast, the downstream signaling pathways Akt/mTOR (p-AKT), RAS/MAPK (p-ERK1/2), and JAK/STAT (p-STAT3) exhibited differential expression across all NB cell lines (**Figure 4C**).

## Discussion

The results of this study underscored the potential of cfDNA WGS as a powerful and noninvasive tool for genomic profiling in NB. By comparing the cfDNA WGS with sequencing on the primary tumor and metastasis at the time of relapse, we demonstrated a high degree of concordance in the identification of key genomic alterations, including *MYCN* and *CDK4/MDM2* amplifications (**Figure 2**; **Supplementary Figure S1B**). These alterations are often associated with aggressive disease and could influence therapeutic strategies (32, 33). These findings highlight the reliability of cfDNA WGS in capturing the genomic landscape of a tumor.

One interesting finding in the cfDNA was the detection of a *MET* p.R970C mutation located in the juxta membrane domain (p.R970C is also known as R988C on transcript NM_001127500.3), which was not identified in both the primary and relapse tumor specimens. MET, a receptor tyrosine kinase (RTK), plays a critical role in tumor growth, invasion, and metastasis. Its activation has been associated with poor prognosis in various cancers (37–41). Other mutations in the juxta membrane of *MET* have been shown to attenuate MET receptor ubiquitination and degradation, thereby prolonging oncogenic signaling (39–42). The *MET* R970C mutation has been previously described in cancer, however with conflicting data on the functionality (43–46). Although our proteomics study revealed MET phosphorylation and activation of the downstream signaling pathways in the resected tumor sample, this activation cannot be directly attributed to the R970C mutation as it was not confirmed in the resected tumor specimen. In addition, these downstream pathways can be activated through other RTKs. This, combined with conflicting data on its oncogenicity and the classification of germline MET R970C as a variant of uncertain significance (VUS) in various ClinVar submissions, highlights the need for further investigations, including functional assays and patient cohort studies, to elucidate its precise involvement in the progression and therapy resistance of NB.

The presence of the novel somatic variants that were not detected in the tumor biopsies emphasizes the advantage of cfDNA in providing a more comprehensive view of tumor heterogeneity and in identifying novel therapeutic targets. Of specific interest in this case is the *MET* mutation that, together with the corresponding protein activation in the tumor tissue (**Figure 4A**), suggests that MET inhibitors could be explored as a targeted therapeutic strategy. The presence of MET activation in a broad range of cell lines (**Figure 4C**), as well as the association between high MET expression and poor survival (**Figure 4B**), indicates that MET could be investigated for therapeutic targeting in patients with NB exhibiting similar genomic and proteomic profiles. Another observation that we identified is the bone marrow metastasis of this NB patient at progressive relapse. In general, *CDK4*/*MDM2*-amplified tumors show minimal bone marrow metastasis (47, 48). The detection of *MET* mutation in cfDNA raises questions about its potential role in tumor progression. In this case, the absence of *MET* mutation in the relapsed tumor sample suggests that this mutation may have been present in sub-clonal populations or was lost during disease progression. Further studies are needed to explore the functional significance of *MET* in NB and its potential association with metastatic behavior.

Altogether, WGS of cfDNA provided a comprehensive overview of the tumor genome of this patient, uncovering a range of somatic mutations and structural variations that are crucial for understanding the biology and progression of the disease. The identification of *MYCN* amplification, along with other significant genomic alterations, highlights the potential of cfDNA WGS as a noninvasive tool for genomic profiling in NB. These findings not only reinforce the concordance with the primary tumor DNA but also uncover additional mutations that may represent sub-clonal populations and inform targeted therapeutic strategies. Due to the risk of circulating tumor DNA contamination in the lymphocyte fraction, there could be limitations to performing somatic analysis of the cfDNA.

In conclusion, this case study highlights the potential of liquid biopsy as a noninvasive tool for the genomic profiling of NB, particularly for the detection of mutations in residual tumors that may be dominated by treatment-resistant clones. One of the limitations in this study is the small sample size, which may have impacted the generalizability of the findings. Larger cohort studies are necessary to validate the observed genomic alterations and their clinical relevance. In addition, while our analysis provides insights into *MET* mutation and its potential role in NB, further functional studies are required to elucidate its precise contribution to tumor progression and metastasis.

## Data Availability

The datasets for this article are not publicly available due to concerns regarding participant/patient anonymity. For this reason, authors of several case reports, recently published in Frontiers journals, have abstained from data deposition in public databases. Requests to access the datasets should be directed to the corresponding author.

## Supplementary material

Supplementary Figure 1
**(A)**. Copy number profiling using the Affymetrix HD SNP microarray shows the patient’s genomic profiles from primary (upper panel) and relapse (lower panel). **(B)**. Coverage plots presented in Integrative genomic visualization (IGV) of tumor and cfDNA sample. Comparison of cfDNA, primary tumor, metastasis, and normal DNA sequencing IGV images of chromosome 2 including *MYCN*, and chromosome 12 covering *CDK4*, and *MDM2*.

Supplementary Figure 2 Overview of number of shared and unique SNV and SV. **(A)** Venn diagram of called non-synonymous SNVs (with variant allele frequency (VAF) ≥10%) and SVs in cfDNA, primary and relapsed tumor. **(B)**. Circos plot of cfDNA showing structural variants, CNVs, and somatic SNVs. Copy number plots are shown on the inner circle, with gain of genomic material indicated in red and loss of genomic material indicated in blue. The lines within the inner circle indicate structural variants within and between chromosomes, while genes affected by somatic SNVs are shown outside the outer circle. Alterations shared between tumor and cfDNA are indicated by red font or red lines, while variants unique to the cfDNA sample is indicated in black.
