# Supplementary figures and images for: Comparative analysis of whole-genome sequencing of tumor and cfDNA in a neuroblastoma patient: a case report
Source: Front Oncol. 2025 May 2;15:1569520. doi: 10.3389/fonc.2025.1569520 (PMC12081241; doi:10.3389/fonc.2025.1569520)

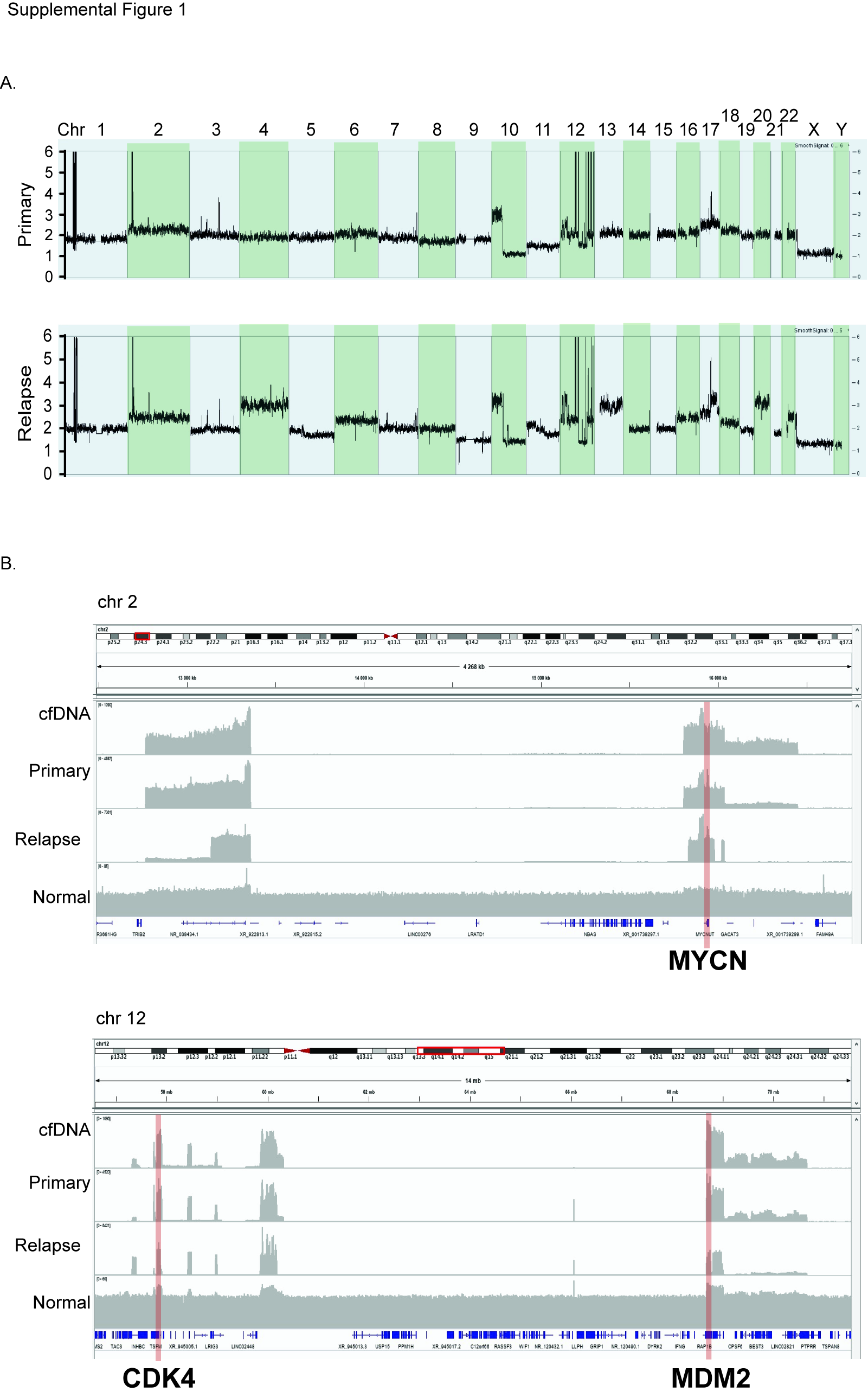

Supplement: Supplementary Figure 1 — (A). Copy number profiling using the Affymetrix HD SNP microarray shows the patient’s genomic profiles from primary (upper panel) and relapse (lower panel). (B). Coverage plots presented in Integrative genomic visualization (IGV) of tumor and cfDNA sample. Comparison of cfDNA, primary tumor, metastasis, and normal DNA sequencing IGV images of chromosome 2 including MYCN, and chromosome 12 covering CDK4, and MDM2. [file Image1.tif]

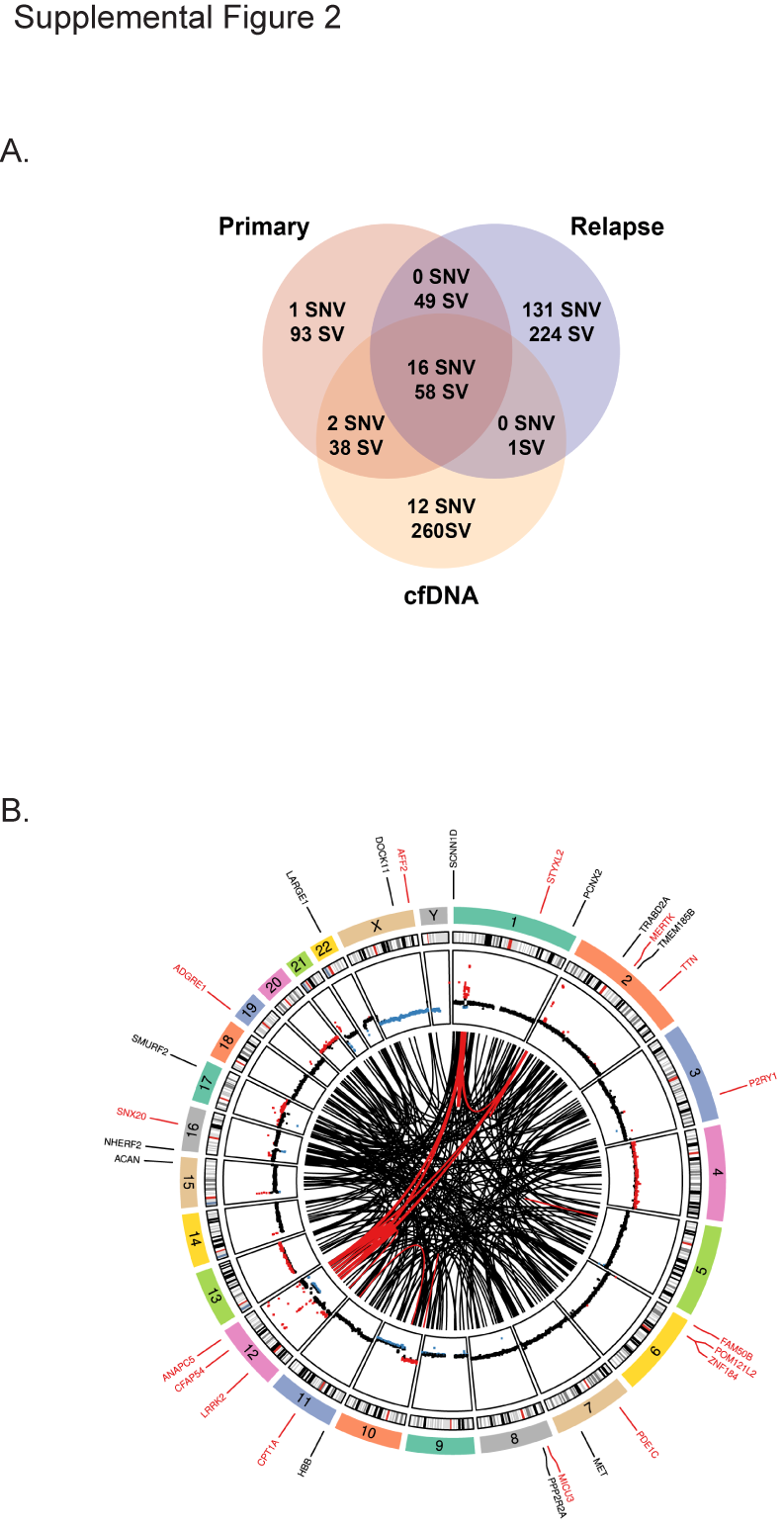

Supplement: Supplementary Figure 2 — Overview of number of shared and unique SNV and SV. (A) Venn diagram of called non-synonymous SNVs (with variant allele frequency (VAF) ≥10%) and SVs in cfDNA, primary and relapsed tumor. (B). Circos plot of cfDNA showing structural variants, CNVs, and somatic SNVs. Copy number plots are shown on the inner circle, with gain of genomic material indicated in red and loss of genomic material indicated in blue. The lines within the inner circle indicate structural variants within and between chromosomes, while genes affected by somatic SNVs are shown outside the outer circle. Alterations shared between tumor and cfDNA are indicated by red font or red lines, while variants unique to the cfDNA sample is indicated in black. [file Image2.tif]
