# Supplementary material for: Comparative analysis of whole-genome sequencing of tumor and cfDNA in a neuroblastoma patient: a case report
Source: Front Oncol. 2025 May 2;15:1569520. doi: 10.3389/fonc.2025.1569520 (PMC12081241; doi:10.3389/fonc.2025.1569520)
Supplement: Supplementary file 3 [file DataSheet1.docx]

**Supplemental Materials and Methods**

**Blood sampling and extraction of cell-free DNA**

Blood samples were collected by clinical nurses at the patients’ regular hospitals, using an existing central venous catheter or peripheral vein puncture. Blood was collected in cf-DNA/cf-RNA Preservative Tubes (Cat. 63950, Norgen Biotech, Thorold, Canada) and plasma was prepared within seven days after collection by centrifugation for 20 minutes at 420 RCF (Heraeus Megafuge 8R, ThermoScientific). cfDNA was extracted from 4 ml plasma using the QIAamp Circulating Nucleic Acid Kit (Qiagen), according to the manufacturer’s instructions with an elution volume of 150 µL. Extracted cfDNA was analyzed with Qubit dsDNA HS Assay Kit (ThermoFisher Scientific) and concentrated using Vivacon 500 centrifugal units with a 30 kDa molecular weight cutoff (Sartorius, Göttingen, Germany), to a final volume of 10–14 µL.

**Whole genome sequencing of DNA from tumor biopsy, blood and cfDNA**

Procedures for WGS performed for tumors with consecutive bioinformatical analyses and filtering procedures have been described in detail earlier [1]. Briefly, paired-end sequencing was performed on Illumina instrumentation (Illumina, San Diego, CA) at Clinical Genomics, The Science for Life Laboratories, Stockholm, Sweden for an average coverage of 59X for primary tumor, 104X for relapsed tumor and 37X for constitutional (normal) DNA. The procedure for sequencing of cfDNA were as follow: Whole-genome sequencing libraries were generated using the NEB Next® Ultra™ DNA Library Prep Kit (Cat No. E7370L). Briefly, cfDNA was processed without further fragmentation or size selection, amplified and ligation with Illumina adapters. The library was evaluated using Qubit and real-time PCR for quantification. Quantified libraries were pooled and sequenced with 150bp paired sequencing on a Novaseq X Plus (Illumina) for an average of 15X, according to the effective library concentration and the data amount required.

Mapping to human reference genome hg38 and somatic and germline variant calling were performed using the Sentieon suite of bioinformatical tool (Sentieon Inc, Mountain View, CA) together with identification of copy number variants through the Canvas tool [2] while somatic structural variants (SV) were called using Manta [3] after systematic filtering and removal of SVs present in SweGen Variant Frequency dataset (https://swefreq.nbis.se/) or in our in-house set of normal controls. Further filtering was done by keeping only high-quality called nonsynonymous variants with coverage above ten, gnomAD allele frequency below 3 % (for somatic variants) and 1% (for constitutional variants) and variant allele frequency equal to or above 10%.

**Genomics profile with SNP microarray**

SNP-Microarray analyses of DNA from tumor were performed using Affymetrix Human Cytoscan High Density arrays essentially as described earlier [19]. For primary data analysis the GDAS software (Affymetrix) was used, while genomic profiles were generated using CNAG (Copy Number Analyzer for AffymetrixGeneChip Mapping arrays) version 3.3 (Genome Laboratory, Tokyo University; http://www.genome.umin.jp; 11).

**Antibodies, cell lines, and reagents**

Phospho-MET (1:1000; Tyr1234/1235; # 3077), MET (1:1000; # 8198), p-STAT3 (1:1000; #9145), p-AKT (1:10,000; #4060), p-ERK1/2 (1:5000; #4370), GAPDH (1:10,000; #5174) antibodies were from Cell Signaling Technology. Horseradish peroxidase (HRP)-conjugated secondary antibody, goat anti-mouse immunoglobulin G (IgG), and goat anti-rabbit IgG (1:10,000) were from Thermo Fisher Scientific. Gastric cell lines (AGS and MKN-45) and neuroblastoma cell lines (Kelly, NB69, SK-N-AS, SK-N-BE (2), and SK-N-FI) were cultured in RPMI 1640 medium with 10% fetal bovine serum and 1% penicillin and streptomycin.

**Immunoblotting**

Gastric cell lines (AGS and MKN-45) and NB cell lines (Kelly, NB69, SK-N-AS, SK-N-BE (2), and SK-N-FI) were used to study the expression/activity of MET. Protein expression and phosphorylation level were investigated by immunoblotting on whole-cell lysates. Cells were lysed on ice with hypotonic lysis buffer [20 mM tris-HCl (pH 7.5), 150 mM NaCl, 1 mM EDTA, 1 mM EGTA, 1% Triton, 2.5 mM sodium pyrophosphate, 1 mM β-glycerophosphate, 1 mM Na3VO4, and leupeptin (1 μg/ml)], with protease/phosphatase inhibitor cocktail (Cell Signaling Technology, #9803) for 15 min and then centrifuged for 10 min at 4°C. Patient tumor sample were disrupted and lysed using the cell lysis buffer in a Tissue Lyser according to the manufacturer’s instruction (Qiagen, #85300). The proteins were separated on 7.5% bis-acryl-tris gels, transferred to polyvinylidene difluoride membranes (Millipore, # IPVH00010), blocked in either 5% bovine serum albumin (phosphoprotein blots) or dry milk (total protein blots) and immunoblotted against primary antibodies overnight at 4°C. Secondary antibodies were diluted 1:10,000 and incubated with shaking at room temperature for 1 hour. Enhanced chemiluminescence substrates were used for detection (Thermo Fisher Scientific, # 34095).

**Kaplan-Meier graph**

Kaplan scanning was performed within R2 (http://r2.amc.nl) on the “Neuroblastoma - Kocak - 649 - custom - ag44kcwolf” dataset, which consists of gene expression data from 649 neuroblastoma tumors that were generated using 44K oligonucleotide arrays. In short, for each gene or other numerical characteristic, R2 calculates the optimal cutoff expression level dividing the patients into a good and a bad prognosis cohort. Samples within a dataset are sorted according to the expression of the investigated gene and divided into two groups on the basis of a cutoff expression value. All cutoff expression levels and their resulting groups are analyzed for survival, with the provision that minimal group number is eight (or any other user-defined value) samples. For each cutoff level and grouping, the log-rank statistical significance of the projected survival is calculated. The best P value and the corresponding cutoff value are selected. This cutoff level is reported and used to generate a Kaplan-Meier graph. The graph depicts the log-rank statistical significance (“raw P”), as well as a P value corrected for the multiple testing (Bonferroni correction) of cutoff levels for each gene.

**Statistical analysis**

Statistical analyses were performed with either GraphPad Prism 7/8 software or R statistical package (v4.0). Statistical tests are indicated in the respective sections and figure captions.

References

1. Fransson, S., et al., *Whole-genome sequencing of recurrent neuroblastoma reveals somatic mutations that affect key players in cancer progression and telomere maintenance.* Sci Rep, 2020. 10(1): p. 22432.

2. Roller, E., et al., *Canvas: versatile and scalable detection of copy number variants.* Bioinformatics, 2016. 32(15): p. 2375-7.

3. Chen, X., et al., *Manta: rapid detection of structural variants and indels for germline and cancer sequencing applications.* Bioinformatics, 2016. 32(8): p. 1220-2.
